# Feeding Infants and Toddlers Study (FITS) 2016: Findings and Thoughts on the Third Data Cycle

**DOI:** 10.1093/jn/nxy158

**Published:** 2018-08-31

**Authors:** Virginia A Stallings

**Affiliations:** 1 Division of Gastroenterology, Hepatology and Nutrition, Children's Hospital of Philadelphia, Philadelphia, PA; 2 Department of Pediatrics, University of Pennsylvania Perelman School of Medicine, Philadelphia, PA

This series of publications in *The Journal of Nutrition* provides a first look at the findings of the 2016 Feeding Infants and Toddlers Study (FITS 2016) and continues the FITS 2002 and FITS 2008 contributions to understanding the diets of very young children. FITS is a national cross-sectional survey of caregivers of infants, toddlers, and young children from birth to <48 mo of age. These contemporary data, collected from June 2015 to May 2016, are based on 3235 households with age-eligible children. FITS is the largest study in the United States to examine dietary intake in this important phase of life for growth and development. Estimates of intake of breast milk, formula, other beverages, foods, and vitamin and mineral supplements are included. The Dietary Guidelines for Americans (DGA) for 2020–2025 are mandated for the first time to include guidelines for children <24 mo of age. The FITS will likely be a major component of the evidence base for the new DGA and along with the DRIs (1), the DGA will provide the needed authoritative sources to guide nutrition-related clinical practice, health monitoring, public health, and public policy recommendations for the youngest children.

## History of the FITS

This is the third cycle of the FITS. The FITS project was launched with national cross-sectional data collected from March to July 2002 for the initial cycle, with 3022 children aged 4 to <24 mo (FITS 2002). The second cycle was collected from June 2008 to January 2009 (FITS 2008) with 3273 children in an expanded age range of birth to <48 mo. This third cycle, collected from June 2015 to May 2016 (FITS 2016), has a sample size of 3235 children, birth to <48 mo of age. The first FITS 2002 findings were published in 2004 as a supplement to the *Journal of the American Dietetic Association* with both commentary and scientific papers (2–11). Reported dietary patterns and estimated nutrient intakes were well-described, and the publications also included detailed analyses of the transition to table food and family meals, and typical snack and meal foods. In 2006, a second FITS 2002 supplement was published with further analyses and commentary (12–25) including details on daily electrolyte intake, portions size, and comparison of Hispanic and non-Hispanic participant dietary intake and feeding practices. The FITS 2008 data and commentary (26–33) were presented in a supplement in 2010 that updated the food and nutrient intake data for US infants, toddlers, and preschool-age children and provided commentary, not only on improvements in intake, but also on the continued concerns and implications of dietary patterns and health. In addition, the authors for FITS 2008 provided key insights and noted evidence gaps that guided the design and goals for the current cycle, FITS 2016.

FITS 2016 expands on the 2 previous cycles by including about 2000 additional foods, as well as new questions designed to address emerging issues in early childhood nutrition and obesity: questions related to modifiable risk factors for obesity (e.g., responsive feeding, which means responding appropriately to infants’ cues to continue or stop feeding), reasons for not breastfeeding infants, food purchasing and preparation habits, children's sleep patterns, child screen time, and household food security. It also reflects a revised food grouping that aligns more closely with the food grouping system used in the NHANES (18).

Among the strengths of FITS over the years are the highly detailed presentation of the data collection and analysis methods used; comparison of the methods with those used over the years in the NHANES; the large, randomized, national samples; and the data collection, management, and analysis being conducted by an independent research organization. FITS 2002 and 2008 contributed evidence for clinical care, monitoring dietary intake, and public health projects designed to improve the health of the youngest US children, and FITS 2016 will continue to provide much needed nutrition evidence in this age group of children who have been scientifically underserved.

## Highlights of FITS 2016

This 2018 supplement is the initial presentation of FITS 2016 as 6 scientific papers and 2 commentaries. As such, it largely presents the results of FITS 2016, and does not include formal evaluation of changes over time in children's intakes with use of the data from all 3 FITS cycles. Because of the changes noted above in the dietary databases and food groupings, such an evaluation, if it is to be valid and statistically sound, requires recoding the 2002 and 2008 data sets to be comparable with the 2016 data set. That work is nearly complete and, once it is, we plan to conduct trend analyses across the 3 cycles and publish the results.

Anater et al. (34) present the FITS 2016 study design and methods in detail and demonstrate that the sample of young children were from representative US households and data were collected and analyzed with sound methods that allow comparison with previous FITS results. The next 2 food intake–based reports divide the 2016 sample by age group, with Roess et al. (35) presenting the findings from participants from birth to 23.9 mo of age and Welker et al. (36) presenting the data from 24 to 47.9 mo of age.

The infant and toddler report (35) examines breastfeeding practices, complementary feeding, and general food consumption patterns. Initiation of breastfeeding has increased modestly since 2008, as has the percentage of infants <9 mo of age who were breastfed at the time of the survey. For infants aged 9–11.9 mo, the percentage currently breastfed has remained stable since FITS 2008. However, non-Hispanic black infants and toddlers were less likely to breastfeed than Hispanic or non-Hispanic white children across the entire range of ages considered in the infant and toddler report. The percentage of all infants consuming vegetables and fruits followed a pattern similar to breastfeeding when compared with FITS 2008: modest increases for infants <9 mo of age and stable for infants aged 9–11.9 mo.

In older children (36), the dietary patterns were examined for those who were 2 y (24–35.9 mo) and 3 y (34–47.9 mo) old. These are ages where young children rapidly change food consumption patterns as they age and are exposed to many meal and snack environments, such as in the home with many food providers (parents, siblings, extended family), day-care, and preschool. Unfortunately, 27% of 2- and 3-y-old children did not consume a distinct vegetable portion in the past 24 h, and as has been reported in previous FITS cycles, of those who did consume a vegetable, fried potatoes were the most common. Another area of concern is that almost all children in this age group consumed a sugar-sweetened beverage, dessert, or sweet or savory snack on the day dietary intake was reported. Almost half consumed a sugar-sweetened beverage, with fruit flavored drinks the most common. Based on these 2 age-based FITS 2016 presentations, we have many opportunities to improve the diets of young children.

Bailey et al. (37) led the analysis of nutrient intake to complement the food and beverage intake results of the previous 2 reports. That paper presents overall nutrient intakes from all foods/beverages (but excluding dietary supplements) and from all foods/beverages plus dietary supplements for all FITS 2016 ages (birth to 47.9 mo) and compares them with the DRI. The paper does not present nutrient intake data for more detailed groups of foods/beverages (e.g., breast milk, foods compared with beverages, major food groups). We hope to publish additional findings on food sources of nutrients in the future.

Of note, the DRI system of nutrient intake recommendations was developed from 1997 to 2005, and only vitamin D and calcium recommendations (38) have been updated since then. It is well accepted that little age-appropriate, high-quality experimental evidence was available to support the Estimated Average Requirement from which the RDA is mathematically derived. In these youngest DRI age groups (0–6 mo, 7–12 mo, 1–3 y) with little high-quality evidence, the DRI relies upon the Adequate Intake process to determine recommendations to support typical growth and health of young children. The Tolerable Upper Intake Level, the component of the DRI that is the highest nutrient intake likely to pose no adverse health risk, also had very little age-appropriate evidence to establish a level for each nutrient intake that may be associated with adverse events. This background is important in considering some of the findings from the analysis (37). A large percentage of young children do not meet the vitamin D or E intake recommendations, and a large percentage exceeded the Upper Limit intake for zinc and vitamin A. There is little evidence that these patterns of average daily intakes of zinc, vitamin A, vitamin D, or vitamin E result in acute or chronic adverse health consequences. Determining the optimal intake of these nutrients should be a research priority so that the clinical and public health recommendations are sufficiently evidence based. We need to determine if these “too high or too low” nutrient intakes pose health risks for young children.

FITS 2016 contributes to understanding the patterns of intake of infants and children enrolled in the Federal Special Supplemental Nutrition Program for Women, Infants, and Children (WIC). WIC provides supplemental food, beverages, and infant formula to qualified families, and currently serves more than half the infants born in the United States. A key FITS 2016 contribution is that it is the first large sample size study to evaluate dietary and nutrient intakes of WIC and non-WIC participating young children since the 2009 WIC food package updates. The WIC program updates harmonized the food package compositions with the current nutrition, food, and child health knowledge and policy. The new WIC program regulations included steps such as eliminating fruit juice from the infant package, additional promotion of breastfeeding initiation and duration, and routine switch to low- or nonfat milk for older children. All of this is summarized in detail in the paper by Guthrie et al. (39) for foods and that by Jun et al. (40) for nutrients. These findings demonstrate opportunities for improvements in dietary intake in WIC participants and to improve the WIC system of nutrition education and supplement foods.

To frame the FITS 2016 contributions, both now and in the near future, Dwyer (41) reviews the strengths of FITS and how this study will strengthen the evidence base for dietary intake and feeding practices of the youngest children in the United States. FITS will support the work of the DGA 2020–2025 and the ongoing NHANES. One hope is that these and other federal programs and evidence-gathering activities will consider the many opportunities identified by FITS 2016 to improve the dietary intake and nutritional status monitoring and policy for this underserved age group of young children.

## Notes

Published in a supplement to *The Journal of Nutrition*. Select contents of this supplement were presented at the Experimental Biology 2017 conference in a session titled, “Informing B-24 Dietary Guidelines: Findings from the New Feeding Infants and Toddlers Study 2016,” held in Chicago, IL, April 22, 2017. Supplement Coordinators were William Dietz, Milken School of Public Health at the George Washington University and Andrea S. Anater, RTI International. The FITS research was funded by Nestlé Research (Lausanne, Switzerland) through a contract with RTI International and its subcontractor, the University of Minnesota. Dr. Dietz is a compensated consultant to RTI on this project. The article contents are the responsibility of the authors and do not necessarily represent the opinions or recommendations of Nestlé. Publication costs for this supplement were defrayed in part by the payment of page charges. This publication must therefore be hereby marked “advertisement” in accordance with 18 USC section 1734 solely to indicate this fact. The opinions expressed in this publication are those of the authors and are not attributable to the sponsors or the publisher, Editor, or Editorial Board of *The Journal of Nutrition*.

Supported by Nestlé Research, Lausanne, Switzerland.

Author disclosures: VAS is a consultant for Medtronics, on the Scientific Advisory Board for Alcresta, AbbVie, and on the Board of Directors for Danone.

Abbreviations used:

DGA: Dietary Guidelines for Americans
FITS 2016: 2016 Feeding Infants and Toddlers Study
WIC: Special Supplemental Nutrition Program for Women, Infants, and Children
